# A Novel Smart Motor Imagery Intention Human-Computer Interaction Model Using Extreme Learning Machine and EEG Signals

**DOI:** 10.3389/fnins.2021.685119

**Published:** 2021-05-06

**Authors:** Yi Gu, Lei Hua

**Affiliations:** School of Artificial Intelligence and Computer Science, Jiangnan University, Wuxi, China

**Keywords:** human-computer interaction model, ELM, motor imagery, EEG signals, BCI data set

## Abstract

The brain is the central nervous system that governs human activities. However, in modern society, more and more diseases threaten the health of the brain and nerves and spinal cord, making the human brain unable to conduct normal information interaction with the outside world. The rehabilitation training of the brain-computer interface can promote the nerve repair of the sensorimotor cortex in patients with brain diseases. Therefore, the research of brain-computer interface for motor imaging is of great significance for patients with brain diseases to restore motor function. Due to the characteristics of non-stationary, nonlinear, and individual differences of EEG signals, there are still many difficulties in the analysis and classification of EEG signals at this stage. In this study, the Extreme Learning Machine (ELM) model was used to classify motor-imaging EEG signals, identify the user’s intention, and control external devices. Considering that single-modal features cannot represent the core information, this study uses a fusion feature that combines temporal and spatial features as the final feature data. The fusion features are input to the trained ELM classifier, and the final classification result is obtained. Two sets of BCI competition data in the BCI competition public database are used to verify the validity of the model. The experimental results show that the ELM model has achieved a classification accuracy of 0.7832 in the classification task of Data Sets IIb, which is higher than other comparison algorithms, and shows universal applicability among different subjects. In addition, the average recognition rate of this model in the Data Sets IIIa classification task reaches 0.8347, which has obvious advantages compared with the comparative classification algorithm. The classification effect is smaller than the classification effect obtained by the champion algorithm of the same project, which has certain reference value.

## Introduction

The brain is the central nervous system that governs human activities. However, in modern society, more and more diseases threaten the health of the brain and nerves and spinal cord, making the human brain unable to conduct normal information interaction with the outside world. In recent years, with the rapid development of computer science and people’s continuous in-depth exploration of brain science, brain-computer interface (BCI) technology has been attracting attention as a new type of human-computer interaction system that directly interacts between brain nerves and peripheral devices. The BCI system collects EEG by placing sensors on the surface of the user’s scalp or inside the skin. The EEG signal is decoded to determine the intention, and then complete a series of operations such as controlling external equipment. Using BCI technology can help patients with movement disorders caused by nerve damage regain the ability to move independently and smoothly interact with the outside world. This technology will significantly improve the quality of life of patients and reduce the burden on families and society. The first international BCI conference held in 1999 clearly defined the brain-computer interface, that is, “BCI is a communication control system for direct communication between the brain and external devices, and does not rely on brain nerves and peripheral muscle tissue” (Wolpaw et al., 2000). Nowadays, BCI technology is developing rapidly at a rapid pace. In the fields of military aviation, rehabilitation and medical treatment, cognitive enhancement, games and entertainment, and intelligence are bursting with strong vitality, the research and application of brain-computer interface has become a hot spot in the field of scientific research (Van Dokkum et al., 2015; Qiu et al., 2017; Hammer et al., 2018).

Motor Imagery (MI) EEG has the characteristics of flexibility, non-invasiveness, low environmental requirements, and high resolution. Therefore, MI is one of the widely used forms of BCI. The frequency band power of the EEG signal during the motion imaging process will vary with the content of the MI task, which is called event related synchronization/desychronization (ERS/ERD). The generation of ERS/ERD is related to internal or external events. When one side of the human limb exercises or performs motor imagination, the energy of μ rhythm and β rhythm in the sensory motor area on the opposite side of the brain decreases, and the energy of μ rhythm and β rhythm in the ipsilateral motor sensory area increases. This rule makes it possible for ERS/ERD to control external equipment or perform motor imagination intention recognition (Lisi et al., 2014). Motor Imagery Brain-Computer Interface (MI-BCI) is used as a branch of the brain-computer interface. The patient’s brain imagines and simulates actions, but there is no actual action output. MI-BCI is mainly based on the analysis and recognition of sensorimotor rhythms, which decodes the signals and converts them into machine instructions. It can establish information channels between humans and machines, and realize the control of devices such as wheelchairs, exoskeletons, and prostheses. According to the theory of neuroplasticity, it also helps to activate nerve cells in a specific area of activity, helping to repair and regenerate damaged nerves.

In 1973, the American Jacques Vidal team put forward the concept of brain-computer interface technology for the first time in a paper, and pointed out that it is an interdisciplinary technology covering various disciplines, including biomedicine, neuroscience, cognitive science, computer science and other fields (Vidal, 1973). However, due to the immaturity of various corresponding technologies at that time, the research of brain-computer interface has always been stagnated at the conceptual and theoretical stage. The scientific research team led by Professor Pfurtscheller in Austria is the pioneer and leader of BCI technology research. The team conducted a lot of research on the BCI system based on the EEG signal of motor imagery, and proposed the concepts of ERD and ERS for the first time (Pfurtscheller, 1992). In 2000, the team developed the Graz-BCI system based on the left and right hand motor imagery EEG signals and the motor sensory region mu and beta rhythm signals, and successfully realized the control of the cursor and the robotic arm (Pfurtscheller et al., 2002). Matsunaga et al. developed a wheelchair control system based on the EEG signal of motion imagination, which can perform basic movement and control operations (Tanaka et al., 2005). In 2008, the research team of the University of Tokyo in Japan used VR technology to conduct feedback training on subjects for the first time, which greatly improved the recognition rate of the subjects’ motor imagination EEG signals (Fujisawa et al., 2008). The Birbaumer laboratory in Germany designed an SCP-based mind converter system. The user can use the EEG signal to control the input of characters, so as to achieve communication with the outside world (Birbaumer et al., 2003). In 2011, a research team from the Technical University of Berlin, Germany, developed a vehicle emergency brake assist system based on EEG signals. This system is 130 ms faster than manual operation and effectively shortens the braking distance (Haufe et al., 2011). In 2015, the Wadsworth Research Center in the United States and Karlsruhe Technical Research Institute in Germany jointly developed the “Brain-to-text” system (Herff et al., 2015). The system uses automatic speech recognition technology to convert human brain activity into corresponding text when speaking. This confirms the possibility of human-computer interaction based on natural speech-related cortical activity. In 2019, the team of Professor Bin He from Carnegie Mellon University in the United States and the University of Minnesota successfully developed the first “non-invasive mind control robotic arm” in history (Edelman et al., 2019). This is a non-invasively connected brain-computer interface system. Users can control the robotic arm to quickly track the randomly moving computer cursor through their mind and imagination. The system has achieved a nearly 60% improvement in learning performance, and the academic performance has also increased by more than five times compared with the previous one. In March 2020, Professor Edward Chang used Recursive Neural Network technology to learn neural features of EEG signals generated by epilepsy patients when they read aloud, and decode them into text sentences. The study achieved 97% accuracy (Makin et al., 2020).

Although China’s sports imagination BCI technology started late, it has also made some progress. The BCI team led by Professor Gao Shangkai of Tsinghua University developed a BCI system in 2007 that uses left and right hand and foot motion imaginary EEG signals to control robotic dogs to complete football kicks (Wang et al., 2007). In 2010, the BCI team of South China University of Technology developed a hybrid BCI system that combined P300 and Mu/Beta rhythms to control the movement of a two-dimensional cursor (Li et al., 2010). In addition, the team used the same method to implement a wheelchair control BCI system in 2012 (Long et al., 2012). In 2015, Xu Baoguo and others from Southeast University developed an online robot control system based on MI-BCI (Xu et al., 2015). On this platform, the feature extraction and classification of the EEG signal of hand movement imagination are realized, and the average classification accuracy rate reaches 91.5%. In 2016, Tang Zhichuan of Zhejiang University and others developed an exoskeleton upper limb rehabilitation robot based on brain control of motor imagination (Tang et al., 2016). The research achieved the highest online classification accuracy rate of 84.29 ± 2.11%. As an effective method to control the exoskeleton of the upper limbs, this research provides a non-invasive brain-controlled active upper limb rehabilitation strategy for clinical applications.

Many experts and scholars have achieved excellent results in the research of brain-computer interface systems and various analysis methods for EEG signals, which has also made a huge promotion for the scientific and effective analysis of EEG signals. Juneja et al. proposed an EEG classification method based on the ELM model, which is characterized by extracting the individual and mutual features of the data set (Juneja and Rana, 2019). Tan et al. proposed an improved ELM model for the classification of synchronous EEG (Tan et al., 2017). Zhang et al. used the differential entropy and attention model to classify EEG, so as to realize the automatic recognition of epilepsy (Zhang et al., 2020). However, the following problems still exist in the practicality of the brain-computer interface. First, the research on the classification of EEG signals for motor imaging mainly focuses on the two-classification problem, which is not enough to meet the actual needs. Second, the algorithm runs for a long time, and it is difficult to meet the user’s requirements for timeliness. Third, the recognition rate needs to be further improved to improve the accuracy of issuing instructions. In response to these problems, this article intends to propose a motor imagery EEG classification method based on ELM algorithm. The main work of this paper is summarized as follows:

(1) The structure and operation process of the MI-BCI system are introduced. The system mainly includes three modules: preprocessing, feature extraction, and classification and recognition. The preprocessing steps in this article include data interception and band-pass filtering. Data interception refers to excluding the preparation and rest phases from the original EEG signal data segment, and only retains the motor imagination phase. Feature extraction includes lead selection, Local Characteristic-scale Decomposition (LCD) feature extraction, and Common Spatial Pattern (CSP) feature extraction. The classification recognition method is the ELM regression model, and the classification is performed by the regression method.
(2) A feature fusion method is used in the feature extraction module. The time domain features obtained based on the LCD feature extraction method and the spatial domain features obtained based on the CSP feature extraction method are combined in series to obtain a fusion feature.
(3) The classic ELM model is used for classification and recognition. The input weight matrix and hidden neuron bias matrix of ELM’s hidden layer is randomly generated and obey any continuous probability distribution, so only the output weight matrix is required to be solved, and the input weight and hidden layer bias need not be adjusted iteratively. Therefore, the calculation amount and time complexity of the algorithm will be much smaller, and the training speed will be much faster. Recognition based on this model is more suitable for tasks that require high response time.

## MI-BCI System Structure

**FIGURE 1:**
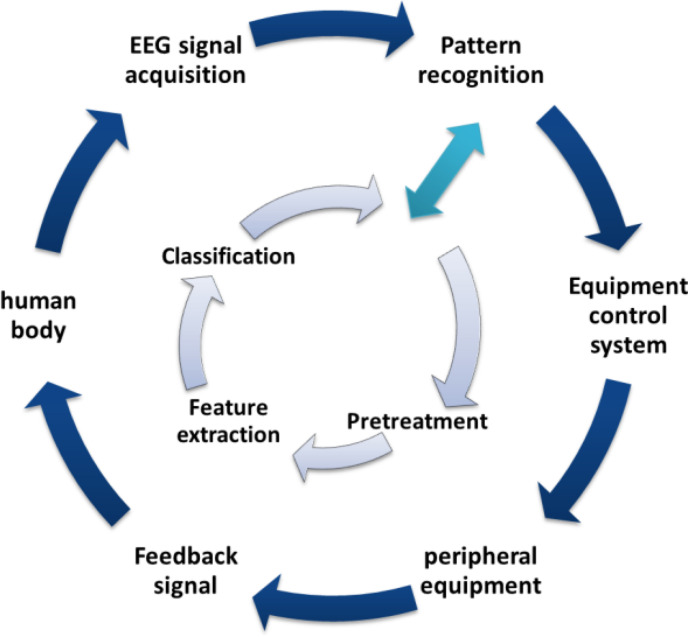
MI-BCI system structure.

The MI-BCI system has different practical applications in different fields and scenarios, but a complete MI-BCI system is basically composed of five parts as shown in Figure 1.

(1) EEG signal acquisition. EEG signal acquisition is mainly responsible for EEG signal acquisition and storage. EEG signal acquisition methods can be divided into two types: non-implantable and implantable. In actual research, non-implantable acquisition methods are used in most cases, that is, the acquisition electrode is directly placed on the corresponding position of the subject’s scalp to record the EEG signal. At the same time, the collected EEG signal is amplified and processed and A/D converted, and the processed result is stored.
(2) Pretreatment. The signal-to-noise ratio of the EEG signal is very low, and it is easily affected by external noise and other biological signals. It is very necessary to preprocess the EEG signal before analyzing and processing it, so as to reduce the artifact interference in the signal and improve the signal quality.
(3) Feature extraction. Feature extraction is one of the most important links in the MI-BCI system. Its purpose is to learn distinguishing features from the preprocessed EEG data, which is the feature that best reflects the true thinking activity of the brain. The commonly used methods include short-time Fourier transform, wavelet packet transform, common space mode algorithm, etc. (Cheng et al., 2019; Hu et al., 2019; Liu et al., 2020; Wang et al., 2020).
(4) Classification and identification. The classification and recognition of EEG signals is one of the key links that determines the performance of the BCI system. The role of this link is to integrate the extracted features and build a classification model. The classification model can establish a mapping relationship between the signal features and the subjects’ real consciousness activities, so as to realize the feature classification of EEG signals. For EEG signals with different characteristics, commonly used classifiers include Fisher discriminant classifier, support vector machine, neural network algorithm, etc. (Rozza et al., 2012; Rubén et al., 2019; Dib et al., 2020; Ghonchi et al., 2020; Luo et al., 2020; Srirangan et al., 2020).
(5) Equipment control system. The function realized by the equipment control system is to convert the classification and recognition results of EEG signals into control commands for peripheral equipment. Common peripheral devices include character input systems, smart wheelchairs, robotic arms, etc.

## ELM-Based Motor Image Recognition Method

### Identification Process

**FIGURE 2:**
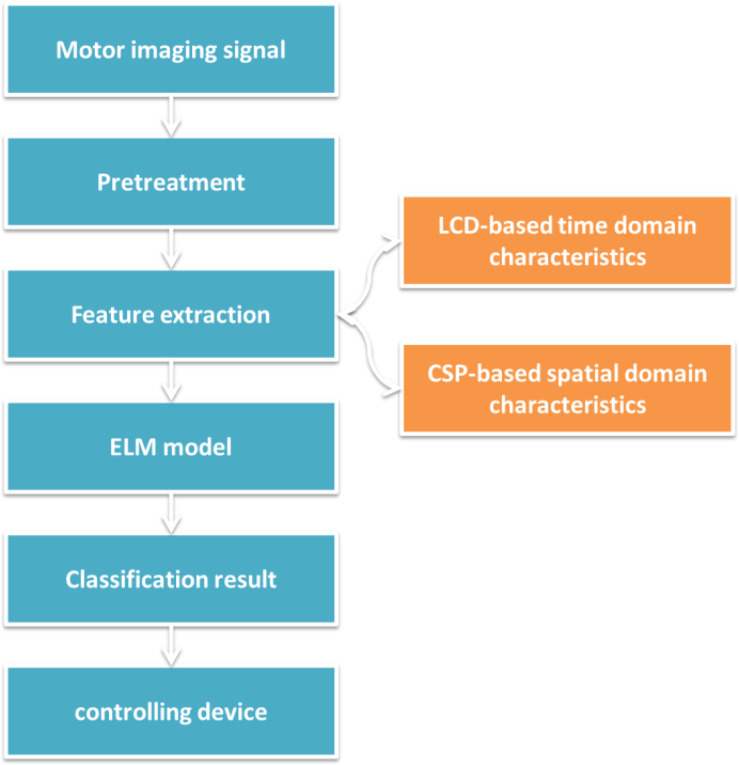
Motion image recognition process based on ELM.

The identification process is shown in Figure 2. First, preprocess the collected motor imaging EEG signals. The preprocessing methods include data interception and band-pass filtering. Data interception removes the original EEG signal data segment from the preparation and rest phases, and only retains the motor imagination phase. Band-pass filtering retains the effective components of the EEG signal in the frequency range of 0.5–30 Hz, and removes irrelevant EMG and ECG components. Second, feature extraction. The time-frequency energy feature and spatial domain feature of EEG signal are extracted, and the features are fused. Third, the extracted features are input into the trained ELM model to obtain the classification result. Fourth, the device is controlled by the mapping relationship between the classification result and the control instruction.

### Feature Extraction

It is necessary to select the lead before feature extraction. The pre-processed signals are calculated and sorted by the contribution rate, and the four leads with the highest information validity are distinguished from the remaining 18 leads. LCD (Yang et al., 2012) is used to decompose four lead EEG signals, 12 intrinsic scale components (ISC) are obtained, and the time-frequency energy feature vector V1 is obtained. To obtain the time-frequency energy of 12 ISC components, the expression of the time-frequency energy is as follows

(1) $$E_{ij}=\sum_{t=0}^{N-1}{\left|ISC_{ij}\left(t\right)\right|}^{2},i=1,2,3,4,j=1,2,3$$

*ISC_ij_*(*t*) refers to the first three layers of ISC components with length N obtained by decomposing each lead. Through the above processing, the time-frequency domain feature vector group *V*_1_ = [H_11_,…,H_13_,…,H_43_] is obtained. CSP (Dornhege et al., 2004) decomposes the remaining 18 leads EEG signals to obtain the spatial domain feature vector V2. A series fusion strategy is adopted to connect V1 and V2. The multiple features are normalized first, and then concatenated end to end. The mathematical expression is as follows:

(2) $$\left\{ \begin{array}{c} V_{1}=\left[\frac{H_{11}}{\left|H_{11}\right|},\ldots ,\frac{H_{13}}{\left|H_{13}\right|},\ldots ,\frac{H_{43}}{\left|H_{43}\right|}\right]\\ V_{2}=\left[\frac{f_{1}}{\left|f_{1}\right|},\frac{f_{2}}{\left|f_{2}\right|},\frac{f_{3}}{\left|f_{3}\right|},\frac{f_{4}}{\left|f_{4}\right|}\right]\\ V=\left[V_{1},V_{2}\right] \end{array}\right.$$

### ELM Classification Model

**FIGURE 3:**
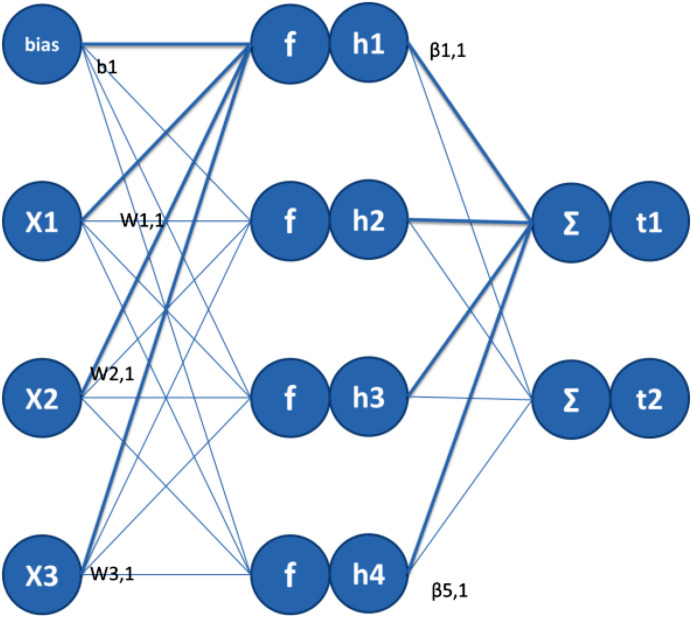
Motion image recognition process based on ELM.

Single hidden layer feed-forward neural networks (SLFN) has been widely used because of its very strong nonlinear approximation ability. The latest research progress on SLFN learning is the Extreme Learning Machine (ELM) algorithm (Tamura and Tateishi, 1997; Huang, 2003; Huang et al., 2004, 2006). ELM is a single-layer feed forward network with fast training. There are only three layers in the network: The Input Layer, the Hidden Layer and the Output Layer. Figure 3 shows the three-tier structure of the network.

In the Input Layer, each sample of each training set will have a corresponding weight and offset. There are two ways in ELM: one is to manually enter these weights and offsets, and the other is to automatically generate weights and offsets through ELM toolbox. Random generation is based on the size of the original data and the sigmoid neuron function. For a linear Output Layer, randomly generated weights can better reflect the performance advantages of ELM. ELM is a regression model, but it is also suitable for classification. If the different categories are separate and independent, create a target for each category separately. The target of the class that can be matched is set to 1, and the target of the unmatched class is set to 0. This encoding creates a unit-length vector for each category. This vector is orthogonal to all other classes of vectors. The distance between the target vectors of different categories is the same, so the independence of the categories can be maintained. The prediction category is assigned based on the target with the largest ELM output.

Literature (Huang et al., 2006) pointed out and proved that if the activation function *g*(*x*) is infinitely differentiable, then, for a given arbitrarily small approximation error, the input layer weights and hidden layer thresholds arbitrarily select the number of hidden nodes *N*′≤*N*, where N is the number of training samples. According to the above theory, when the activation function *g*(*x*) is infinitely differentiable, all network parameters do not need to be adjusted. The input weight *w_i_* and the hidden layer threshold *b_i_* can be randomly assigned during training, and they are fixed during the training process. For the selected sample data, the hidden layer output matrix is fixed. Therefore, the parameter training of ELM is transformed into solving the following linear regression problem:

(3) $$f\left(x\right)=\beta^{T}g$$

Here, β can be obtained by solving the least squares solution of the linear system *H*β = *T*. For the multiple-input single-output data set *D* = {(*x_n_*,*t_n_*)|*x_n_*∈*R^p^*,*t_n_*∈*R,n* = 1,2,…,*N*.}, the following optimization problems can be solved:

(4) $$\min_{\beta }||H\beta -T||$$

where $H={\left[\begin{array}{ccc} g(w_{{}_{1}}^{T}x_{1}+b_{1}) & ... & g(w_{{}_{N^{\prime }}}^{T}x_{1}+b_{N^{\prime }})\\ ... & ... & ...\\ g(w_{{}_{1}}^{T}x_{N}+b_{1}) & ... & g(w_{{}_{N^{\prime }}}^{T}x_{N}+b_{N^{\prime }}) \end{array}\right]}_{N\times N^{\prime }}={\left[\begin{array}{c} g^{T}\left(x_{1}\right)\\ ...\\ g^{T}\left(x_{N}\right) \end{array}\right]}_{N\times N^{\prime }}$ is the hidden output matrix. β = [β_1_,β_2_,…,β_*N*′_]^*T*^ is the output node, *T* = [*t*_1_,*t*_2_,⋅⋅⋅,*t_N_*]^*T*^ is the hidden layer connection weight vector. The least square solution of Eq. (3) is

(5) $$\hat{\beta }=H^{+}T$$

where *H*^+^ is the Moore-Penrose generalized inverse of the hidden output matrix *H*. The optimal solution A has the following important characteristics (1) The smallest training error can be obtained through this solution; (2) The weight vector of the smallest normal form is obtained; (3) The least squares solution of the paradigm is unique, so the algorithm will not produce a local optimal solution.

## Experimental Process and Results Analysis

### Experimental Data Set

The data set used in this article is the BCI competition data set provided by Graz University. The description of the experimental data is shown in Table 1.

**TABLE 1:** Data set introduction.

Data set	Subjects	Tasks	Channels	Rate	Experiments	Years
Data Sets IIb (Michael et al., 2012)	9	2 (left hand right hand)	3	250	400	2008
Data Sets IIIa (Schlögl et al., 2005)	3	4 (Left hand, right hand, tongue, feet)	60	250	240	2005

### Experimental Setup

To verify the effectiveness of the method used in this paper, the selected contrast classifiers include support vector machine SVM (Shahyad et al., 2019), Bayesian classifier (Bayesian) (Zhang et al., 2016), Takagi–Sugeno–Kang (TSK) (Gu et al., 2017). To verify the superiority of the fusion features, the individual time-frequency features and spatial domain features were compared with the fusion features of the two in the experiment process. This paper uses the classification accuracy rate acc and the kappa coefficient to measure the classification accuracy to evaluate the classification performance of the model. The larger the kappa coefficient, the better the algorithm performance. The mathematical expression of the kappa coefficient is as follows:

(6) $$Kappa=\frac{acc-1/N}{1-1/N}$$

where *acc* represents the classification accuracy rate, and N represents the number of categories. The value range of kappa is [0,1]. The larger the value, the stronger the consistency and the higher the classification accuracy. During the experiment, the data set was divided into training set and test set. The training set is used to build the classifier model, and the test set is used to evaluate the accuracy of the model for predictive classification of unknown samples. To scientifically test the classification accuracy rate and avoid misleading the experimental results caused by a single classification result, this article adopts fivefold cross validation. Each time, the data set is divided into five parts, and four of them are used as training data to obtain the model completely randomly. The remaining one is classified as test data. The trained classifier model is used to classify the test set. From the classification results, the accuracy rate *acc*_*i*_ (i = 1, 2,…, 5) and the Kappa coefficient *K*_*i*_ (i = 1, 2,…, 5) can be obtained. The above process is repeated five times, and the average value of five times is used as the final accuracy of the algorithm. The final calculation formula of the experimental results is as follows, where *F* is 5.

(7) $$\bar{A}=\frac{1}{F}\sum_{i=1}^{F}acc_{i}$$

(8) $$\bar{K}=\frac{1}{F}\sum_{i=1}^{F}K_{i}$$

### Discussion of Experimental Results

(1) Data Sets IIb

**FIGURE 4:**
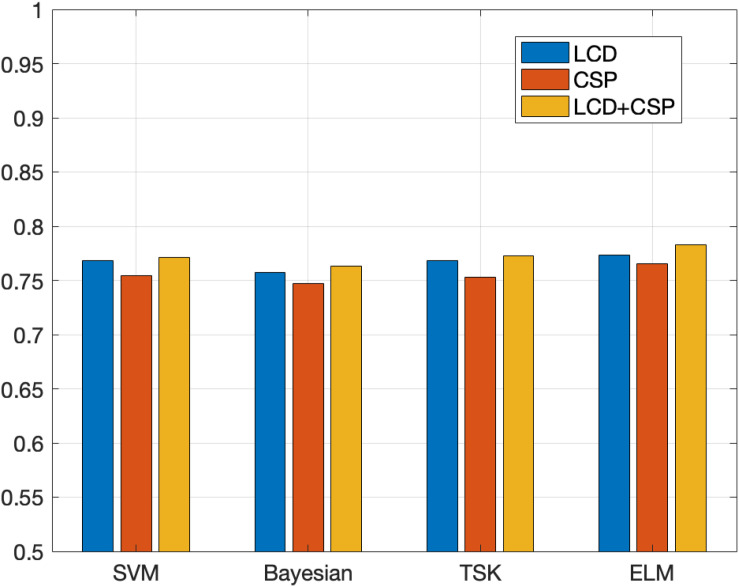
Classification accuracy obtained by each classification model on the Data Sets IIb.

**FIGURE 5:**
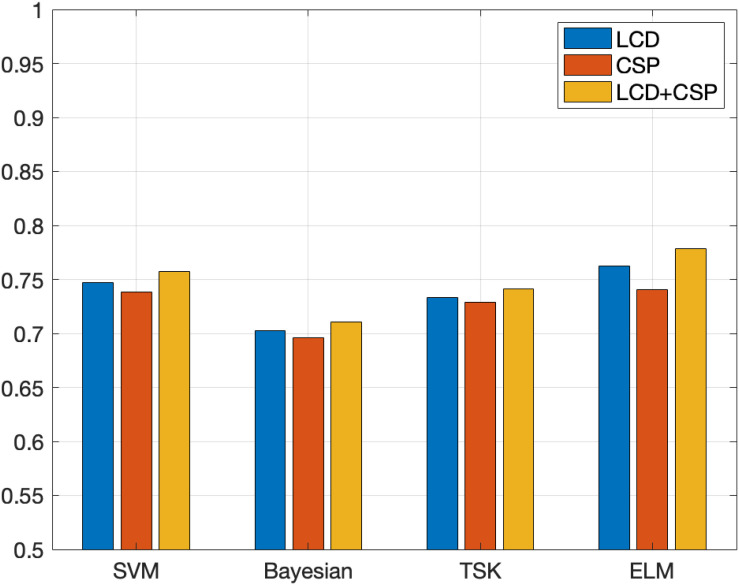
Kappa coefficient obtained by each classification model on the Data Sets IIb.

In order to demonstrate the effectiveness of the method in this paper, this paper compares feature extraction and classifier performance. The experimental results on the Data Sets IIb data set are shown in Tables 2, 3. Figures 4, 5 show the classification accuracy and Kappa coefficient obtained by each classification model on the Data Sets IIb data set. The classification on this data set is a binary classification problem.

**TABLE 2:** Classification accuracy of data sets IIb.

Subject	Feature extraction	SVM	Bayesian	TSK	ELM
1	LCD	0.7374	0.7288	0.7392	0.7408
	CSP	0.7286	0.7074	0.7111	0.7394
	LCD+CSP	0.7421	0.7320	0.7447	0.7532
2	LCD	0.6879	0.6724	0.6854	0.6858
	CSP	0.6580	0.6693	0.6622	0.6741
	LCD+CSP	0.7002	0.6798	0.6905	0.6998
3	LCD	0.7896	0.7669	0.7987	0.8008
	CSP	0.7744	0.7602	0.7880	0.7983
	LCD+CSP	0.7901	0.7754	0.8022	0.8112
4	LCD	0.7417	0.7491	0.7488	0.7504
	CSP	0.7289	0.7352	0.7306	0.7422
	LCD+CSP	0.7498	0.7594	0.7523	0.7610
5	LCD	0.7835	0.7713	0.7864	0.7915
	CSP	0.7749	0.7645	0.7800	0.7798
	LCD+CSP	0.7906	0.7802	0.7939	0.8003
6	LCD	0.7965	0.7843	0.7892	0.8002
	CSP	0.7893	0.7748	0.7816	0.7918
	LCD+CSP	0.7998	0.7841	0.7889	0.7988
7	LCD	0.8117	0.7993	0.7943	0.8016
	CSP	0.8023	0.7892	0.7868	0.7994
	LCD+CSP	0.8108	0.8034	0.7979	0.8180
8	LCD	0.7952	0.7867	0.8032	0.8128
	CSP	0.7794	0.7772	0.7804	0.7950
	LCD+CSP	0.7864	0.7893	0.8089	0.8236
Avg	LCD	0.7679	0.7574	0.7682	0.7730
	CSP	0.7545	0.7472	0.7526	0.7650
	LCD+CSP	0.7712	0.7630	0.7724	0.7832

**TABLE 3:** Kappa coefficient of Data Sets IIb.

Feature extraction	SVM	Bayesian	TSK	ELM
LCD	0.5017	0.4841	0.5208	0.5619
CSP	0.4986	0.4749	0.5004	0.5572
LCD+CSP	0.5135	0.4981	0.5337	0.5897

The experimental results on Data Sets II give two feedback points. (1) The three features are the time domain features based on the LCD feature extraction method, the spatial domain features based on the CSP feature extraction method, and the fusion feature obtained by combining the time domain feature and the spatial domain feature. The classification results obtained based on the above three features show that the classification effect obtained by fusing the features is the best. Different feature extraction methods perform basically the same on the four classification algorithms. For each classifier, the classification effect based on fusion features is the best. This shows that the information of fusion features is richer and more comprehensive, which is more conducive to classification tasks. In most classification algorithms, the classification effect based on the LCD feature extraction method is better than the classification effect based on the CSP feature extraction method. This shows that time domain features have higher information value than spatial domain features. (2) Comparing the four classification algorithms, the ELM algorithm used in this article has the best performance, followed by TSK, third is SVM, and the worst is Bayesian. For other types of classification algorithms, ELM can initialize the input weights and biases randomly to get the corresponding output weights. This algorithm can be faster than traditional learning algorithms under the premise of ensuring learning accuracy. This is the reason why this algorithm is chosen as the classification model in this article.

(2) Data Sets IIIa

**FIGURE 6:**
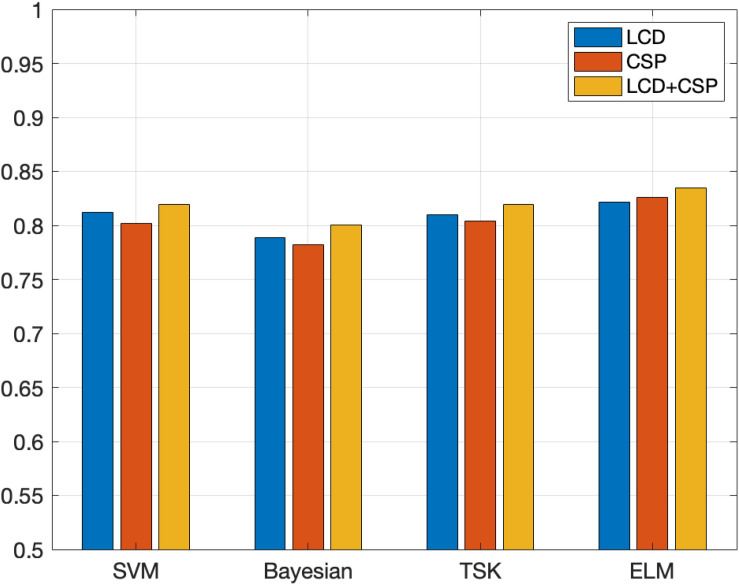
Classification accuracy obtained by each classification model on the Data Sets IIIa.

**FIGURE 7:**
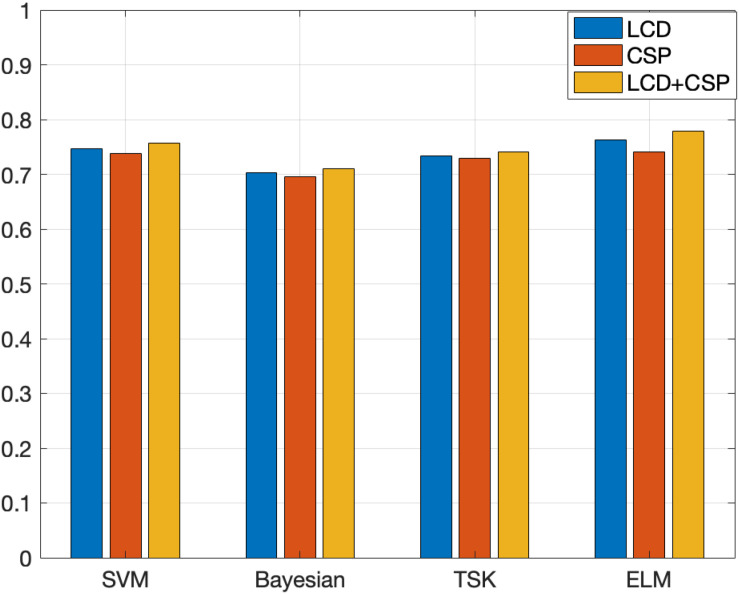
Kappa coefficient obtained by each classification model on the Data Sets IIIa.

The classification results on the Data Sets IIIa data set are shown in Tables 4, 5. Figures 6, 7 show the classification accuracy and Kappa coefficient obtained by each classification model on the Data Sets IIIa data set. The classification of this data set belongs to four classifications.

**TABLE 4:** Classification accuracy of data sets IIIa.

Subject	Feature extraction	SVM	Bayesian	TSK	ELM
1	LCD	0.8257	0.8031	0.8209	0.8286
	CSP	0.8135	0.8059	0.8187	0.8145
	LCD+CSP	0.8294	0.8202	0.8231	0.8466
2	LCD	0.8121	0.7985	0.8073	0.8252
	CSP	0.8093	0.7917	0.8028	0.8565
	LCD+CSP	0.8289	0.8123	0.8162	0.8334
3	LCD	0.7982	0.7645	0.8014	0.8105
	CSP	0.7819	0.7492	0.7906	0.8067
	LCD+CSP	0.7998	0.7685	0.8182	0.8242
Avg	LCD	0.8120	0.7887	0.8099	0.8214
	CSP	0.8016	0.7823	0.8040	0.8259
	LCD+CSP	0.8194	0.8003	0.8192	0.8347

**TABLE 5:** Kappa coefficient of data sets IIIa.

Feature extraction	SVM	Bayesian	TSK	ELM
LCD	0.7472	0.7022	0.7331	0.7623
CSP	0.7380	0.6958	0.7290	0.7405
LCD+CSP	0.7572	0.7105	0.7412	0.7788

The data set on Data Sets IIIa is classified into four categories, and the experimental results obtained are somewhat different from the experimental results on Data Sets II. By analyzing the experimental data in Tables 4, 5, the following conclusions are obtained. (1) The fusion feature extraction method among the three feature extraction methods has the best classification effect. On this data set, the classification effect based on the LCD feature extraction method is better than the classification effect based on the CSP feature extraction method, but the gap in classification accuracy is not very large. (2) The classification effect of the ELM algorithm is still the best, followed by SVM, TSK third, and Bayesian the worst. Compared with the experimental results obtained from the Data Sets II data set, the position of the best classification effect of ELM has not changed, but the classification effect of SVM classifier surpasses TSK. This fully demonstrates the feasibility and superiority of selecting ELM as the classification algorithm in this paper. Compared with other classification algorithms, the classification performance of the ELM algorithm is more stable and the advantages are more obvious.

## Conclusion

A novel smart motor imagery intention human-computer interaction model using extreme learning machine and EEG signals is proposed. Aiming at the problems of low recognition accuracy, large individual differences, and feature redundancy in the current multi-classification problem of motor imagery EEG, this paper deeply studies the signal recognition methods of different limb motor imagery to improve the application of BCI system. The main work of this paper is summarized as follows: (1) A multi-feature fusion extraction method, namely the feature extraction method of LCD+CSP, is used. This method can extract the time-frequency domain and spatial domain features of the data set. The experimental results show that the classification result obtained by a single feature is not as good as the classification result obtained by the fusion feature. (2) The selected ELM algorithm is a mature and widely used classification algorithm with relatively high classification accuracy and fast running speed. ELM can initialize the input weights and biases randomly to get the corresponding output weights. This algorithm can be faster than traditional learning algorithms under the premise of ensuring learning accuracy. The experimental results verify that the ELM algorithm has certain classification advantages. The motion image intention recognition effect based on the method in this paper is close to the champion algorithm of BCI competition. Therefore, the human-computer interaction model designed based on the recognition results of this method is feasible to help patients recover. However, the data set used in this article is relatively simple, and they are all data sets in the BCI competition. In the follow-up, this research plans to use richer data sets for experimental research to verify the universality of the algorithm in this paper. The key to the effectiveness of the ELM model is whether it is necessary to extract features from the data. If feature extraction is meaningless, ELM can come in handy. If it is for the original data set, and feature extraction is very important, the classification effect of ELM is not ideal. In addition, ELM sacrifices too many meaningful patterns in data for speed. Therefore, how to improve ELM to determine the balance between classification accuracy and speed is a future research work.

## Data Availability Statement

Publicly available datasets were analyzed in this study. This data can be found here: http://www.bbci.de/competition/iv/ and http://www.bbci.de/competition/iii/.

## Author Contributions

YG developed the theoretical framework and model in this work and drafted the manuscript. YG and LH implemented the algorithm and performed experiments and result analysis. Both authors contributed to the article and approved the submitted version.

## Conflict of Interest

The authors declare that the research was conducted in the absence of any commercial or financial relationships that could be construed as a potential conflict of interest.
